# Enhanced expression of LINE-1-encoded ORF2 protein in early stages of colon and prostate transformation

**DOI:** 10.18632/oncotarget.6767

**Published:** 2015-12-26

**Authors:** Chiara De Luca, Fiorella Guadagni, Paola Sinibaldi-Vallebona, Steno Sentinelli, Michele Gallucci, Andreas Hoffmann, Gerald G. Schumann, Corrado Spadafora, Ilaria Sciamanna

**Affiliations:** ^1^ Istituto Superiore di Sanità, SBGSA, Rome, Italy; ^2^ Laboratory BioDAT SR Research, IRCCS San Raffaele Pisana, Rome, Italy; ^3^ Department of Experimental Medicine and Surgery, University “Tor Vergata”, Rome, Italy; ^4^ I.F.O. Regina Elena, UOC Pathological Anatomy/Urology, Rome, Italy; ^5^ Department of Medical Biotechnology, Paul-Ehrlich-Institut, Langen, Germany; ^6^ Institute of Translational Pharmacology, CNR, Rome, Italy

**Keywords:** retrotransposon, LINE-1/L1, ORF2, reverse transcriptase, tumorigenesis

## Abstract

LINE-1 (L1) retrotransposons are a source of endogenous reverse transcriptase (RT) activity, which is expressed as part of the L1-encoded ORF2 protein (L1-ORF2p). L1 elements are highly expressed in many cancer types, while being silenced in most differentiated somatic tissues. We previously found that RT inhibition reduces cell proliferation and promotes differentiation in neoplastic cells, indicating that high endogenous RT activity promotes cancer growth. Here we investigate the expression of L1-ORF2p in several human types of cancer.

We have developed a highly specific monoclonal antibody (mAb chA1-L1) to study ORF2p expression and localization in human cancer cells and tissues.

We uncover new evidence for high levels of L1-ORF2p in transformed cell lines and staged epithelial cancer tissues (colon, prostate, lung and breast) while no or only basal ORF2p expression was detected in non-transformed cells. An in-depth analysis of colon and prostate tissues shows ORF2p expression in preneoplastic stages, namely transitional mucosa and prostate intraepithelial neoplasia (PIN), respectively.

Our results show that L1-ORF2p is overexpressed in tumor and in preneoplastic colon and prostate tissues; this latter finding suggests that ORF2p could be considered as a potential early diagnostic biomarker.

## INTRODUCTION

Long Interspersed Element-1 (LINE-1, L1) members belong to the family of autonomous retrotransposable elements (or retrotransposons), which spread in the human genome via RNA intermediates. L1 retrotransposons comprise about 5 × 10^5^ copies collectively accounting for about 17% of the genome [1]. Each functional L1 member includes two open reading frames, ORF1 and ORF2, expressed as a bicistronic RNA. ORF1 and ORF2 encode a 40-kDa RNA-binding protein (ORF1p) and a 150-kDa polyprotein (ORF2p), respectively; the latter includes an N-terminal endonuclease (EN) and a reverse transcriptase (RT) domain [2]. Only 80–100 human L1 copies are full-length and retrotransposition-competent [3], whereas the majority of L1 insertions is truncated at their 5′ end and thus not mobile [4]. Therefore, most genomic L1 elements cannot retrotranspose, yet are transcriptionally proficient and could provide a source of EN and RT activities [5]. It has been shown that induction of L1 expression in normal human cells promotes a senescence-like phenotype [6] and that both the EN and RT domains of L1-ORF2p can individually affect viability of cancer cells [7]. To avoid harmful consequences to the host genome due to uncontrolled L1 expression and mobilization, several molecular mechanisms have been developed to repress retrotransposon activity in healthy somatic cells [reviewed in 8]. Among those, DNA methylation exerts a broad repressive effect by creating non-permissive contexts for L1 function [9, 10]; in contrast, hypomethylation triggers retrotransposon reactivation [11], with the ensuing dysregulation of a variety of genome functions frequently associated with the insurgence of cancer [12, 13]. In the past years, we have investigated the function of L1-encoded RT in tumorigenesis using two complementary approaches: in the first one, we inhibited the RT activity pharmacologically in tumor cell lines using efavirenz, a non-nucleoside RT inhibitor used in AIDS therapy [14, 15]; in the second approach, we downregulated the expression of L1-encoded ORF2p by RNA interference (RNAi) [14, 16]. Both approaches consistently reduced proliferation and restored differentiation traits in cancer cells, yielding remarkable changes in cell morphology and in global transcription profiles of both coding and non-coding RNAs [15]; notably, neither approach caused substantial alterations in non-transformed cells.

Furthermore, both RNAi-mediated downregulation of L1 expression [16] and the administration of efavirenz [14] drastically reduced the tumorigenic potential of tumor cells xenografted in nude mice, thus demonstrating a therapeutic efficacy *in vivo* in preclinical models. These findings were independently confirmed in several other laboratories after treating human cancer cell lines with both nucleoside [17–19] and non-nucleoside [20–23] RT inhibitors. Finally, the outcome of a phase II clinical trial of prostate metastatic carcinoma patients treated with efavirenz indicates that L1-encoded RT can be regarded as a potential therapeutic target in a novel cancer differentiation therapy [24].

We have recently proposed a model based on the central role of L1 RT in governing the balance between single- and double-stranded RNAs, through the formation of RNA:DNA hybrids. This mechanism hypothesizes that in cancer cells the generation of RNA:DNA hybrid molecules “subtracts” templates for double-stranded RNA formation and hence impairs the production of regulatory miRNAs, with a global alteration of gene expression [15, 25].

Consistent with the idea that L1 RT is a key player in tumorigenesis, we recently found that ORF2p expression increases early at cancer onset in a transgenic murine model of breast cancer [26]. This finding is consistent with other group's results showing that ORF1p is also detected in various human cancers [27] and that L1 products are generally highly expressed in breast [28, 29], gastric [30, 31] and pediatric germ cell tumors [32], but not in their healthy tissue counterparts. Moreover, nuclear localization of L1 proteins is associated with a poor prognosis in breast cancer [28, 29], suggesting that compartmentalization in different subcellular domains correlates with different biological roles.

Most studies, aiming at the characterization of L1 proteins in human cancer tissues, have used antibodies directed against L1-ORF1p [27, 28], with only one report [29] making use of an anti-ORF2p polyclonal antibody. In spite of the data summarized above, suggesting a key mechanistic implication of L1 RT in tumorigenesis, the protein itself has been difficult to study in human cancer due to the poorly reliable performances of currently available antibodies. We have thus developed a suitable reagent to ensure accurate detection of ORF2p. We report the development of a novel, highly sensitive monoclonal antibody (mAb chA1-L1) targeting ORF2p and its use in human cancer cell lines and bioptic samples. We demonstrate that ORF2p is enhanced in several human cancer tissues among which colon and prostate show high level of ORF2p expression at very early stages of transformation, well before the appearance of cancer-typical histological alterations.

## RESULTS

### Production and validation of a new monoclonal antibody against L1-encoded ORF2p

**Figure 1:**
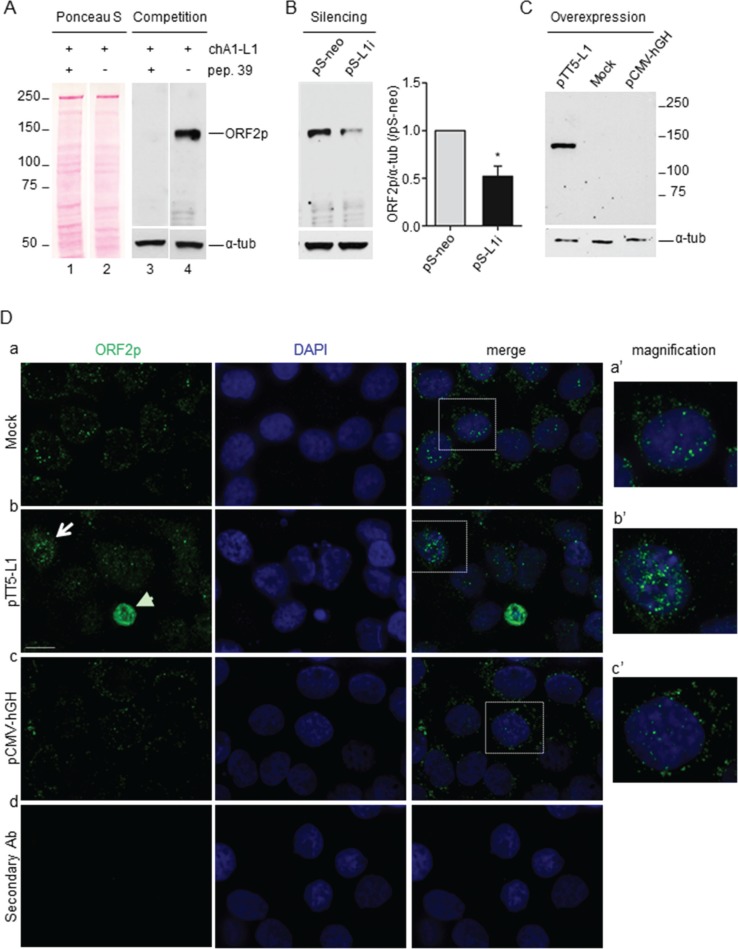
chA1-L1 monoclonal antibody specifically recognizes both endogenous and overexpressed ORF2p in A-375 melanoma cells (**A**) Peptide competition assay. Whole cell extract (50 μg/lane) from A-375 melanoma cells were stained with Ponceau S (lanes 1 and 2); filters were incubated with chA1-L1 mAb pre-incubated with peptide 39 (lane 3) or mAb alone (lane 4). chA1-L1 mAb identifies a single band at the predicted migration for ORF2p molecular mass, 150 kDa; peptide 39 abrogates the binding of chA1-L1 with the antigen; α-tubulin served as loading control. (**B**) Immunoblot analysis of whole cell extract (50 μg) from A-375 cells interfered with vector (pS-neo, control), or with L1-interfering shRNAs (pS-L1i), using chA1-L1 antibody; α-tubulin is used as a loading control. Histograms represent the densitometric quantification of band signal intensities; data are shown as fold change relative to control (pS-neo) after normalization to α-tubulin. Data are expressed as mean ± S.D. of three independent experiments; *P < 0.05 (paired *t* test). (**C**) Immunoblot analysis of cell extract from pTT5-L1-, mock- and pCMV-hGH-transfected A-375 cells using chA1-L1 mAb to detect transiently overexpressed ORF2p. Notably, 5 μg of cell extract (i.e., 10-fold less than in A and B) were loaded on the gel; α-tubulin served as loading control. (**D**) Immunofluorescence assay of mock- (a), pTT5-L1- (b) and pCMV-hGH-transfected (c) A-375 cells. Cells were stained with chA1-L1 mAb and DAPI to detect ORF2p (green) and cell nuclei (blue), respectively. chA1-L1 mAb is omitted in the negative control (d). Bar, 10 μm; magnification 100x. Higher magnification of the boxed areas in (a′), (b′) and (c′).

Aiming to analyze the expression of ORF2p in cancer, we produced a mouse monoclonal antibody against the human L1_RP_-ORF2p [33]. BALB/c mice were immunized separately with six (see Materials and Methods) human L1-ORF2p-derived peptides (#39–44); peptide 39, contained in the EN domain, was identified as the most immunogenic by immunoblot and ELISA assays of mice sera (Supplementary Figures S2 and S3) and a hybridoma cell line was established. The released monoclonal antibody (mAb chA1-L1) was tested for its ability to specifically recognize ORF2p in A-375 human melanoma cells, which are known to express L1-ORF2p [16], using three independent criteria (Figure 1). First, in a peptide competition assay (Figure 1, panel A), the pre-incubation of chA1-L1 antibody with peptide 39 abrogates the binding of the antibody with its 150 kDa cognate protein (lane 3), which is instead clearly depicted when the peptide was omitted (lane 4). Second, as shown in panel B, the band intensity was significantly reduced when mAb chA1-L1 was tested on extracts from A-375 cells in which L1 expression was stably downregulated by RNA interference (RNAi) (lane pS-L1i), in comparison with non-interfered control cells (lane pS-neo) [16]. Third, mAb chA1-L1 quantitatively detected ORF2p expression in A-375 cells transiently transfected with the L1-ORF2p expression plasmid pTT5-L1 (Figure 1, panel C) (Supplementary Figure S1) thus confirming its high specificity (see also Supplementary Figure S4). The sensitivity of the monoclonal antibody chA1-L1 in ORF2p detection was assessed by immunoblot detection of increasing amounts of purified L1-EN protein [34] (Supplementary Figure S5). This assay confirmed the sensitivity of the antibody detecting as little as 3 ng of L1-EN. To assess whether mAb chA1-L1 also recognizes L1-ORF2p in its native conformation, we performed immunofluorescence (IF) experiments using A-375 cells transfected with either pTT5-L1, or pCMV-hGH expressing human growth hormone (hGH) [35] or mock-transfected. As shown in Figure 1D, rows a-c, mAb chA1-L1 yielded punctuated IF signals in both nuclei and cytoplasms. In pTT5-L1-transfected cells, that transiently overexpress ORF2p, a more intense staining was appreciated (row b, arrow); a subset of pTT5-L1-transfected cells displayed small pyknotic nuclei, suggestive of cell death, associated with intense cytoplasmic staining (arrowhead); indeed, in double IF staining assays, we found that cells with abundant cytoplasmic ORF2p staining concomitantly expressed caspase-3 (data not shown), suggesting that an excess of exogenous ORF2p is toxic to the cells, which is consistent with a previous report [36]. No IF signals were observed in pTT5-L1-transfected A-375 cells incubated with secondary antibody alone (row d).

**Figure 2:**
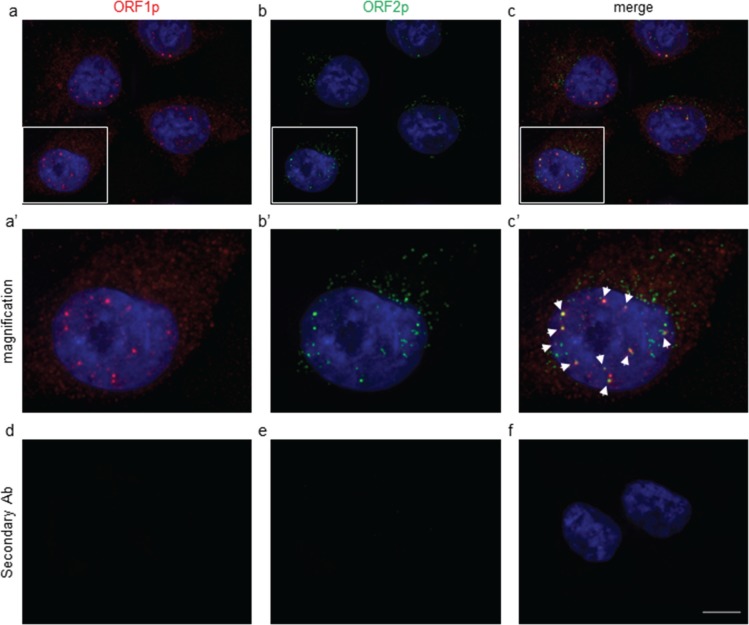
Immunofluorescence assay of A-375 cells stained with anti-ORF1p polyclonal (red, a) and chA1-L1 anti-ORF2p monoclonal (green, b) antibodies DAPI was used to detect cell nuclei (blue). Both primary antibodies were omitted in the negative control (d, e, f). Bar, 10 μm; magnification 100x. Higher magnification of the boxed areas in (a′), (b′) and (c′); white arrowheads in (c′) indicate colocalization foci of ORF1 and ORF2 proteins.

It is known that L1-encoded ORF1 and ORF2 proteins preferentially associate with their encoding transcript to form ribonucleoprotein particles (RNPs) [37]. To further confirm the mAb specificity and to compare the ORF1p and ORF2p staining patterns on melanoma cells, we performed a double IF assay using chA1-L1 mAb and a polyclonal anti-ORF1p antibody [38]. As shown in Figure 2, endogenous ORF1 and ORF2 proteins are detected both in nuclei and cytoplasms (panel a, a′ and b, b′, respectively); interestingly, the staining indicates colocalization of L1-ORF1 and -ORF2 proteins in the nucleus (panels c, c′), thus confirming the close association of both proteins. No IF signals are detected when the primary antibodies are omitted (panels d, e, f). The identification of endogenous L1-ORF2p in the nucleus is consistent with the model of L1-mediated target-primed reverse transcription [39, 40] and with previous reports demonstrating nucleolar [41] and nuclear [42] localizations of ORF2p and ORF1p, respectively.

### ORF2p is expressed in a variety of human tumorigenic cell lines

**Figure 3:**
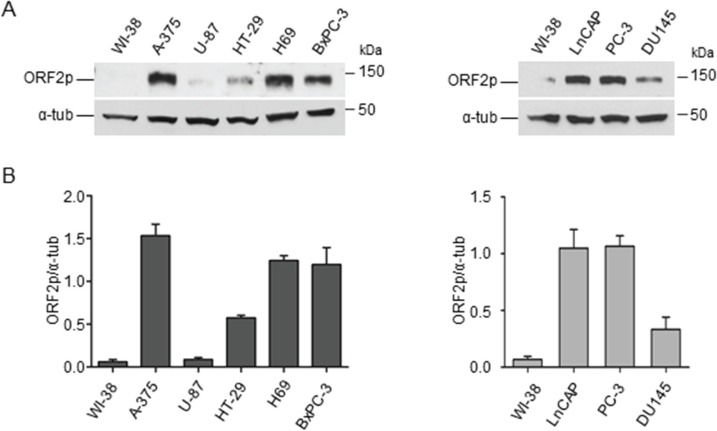
Detection of ORF2 protein in human cancer cell lines (**A**) Immunoblotting analysis of ORF2p in the indicated cell lines. WI-38, normal fibroblasts; A-375, melanoma; U-87, glioblastoma; HT-29, colon carcinoma; H69, small cell lung carcinoma; BxPC-3, pancreas carcinoma; LnCAP, PC-3 and DU145, prostate carcinoma cell lines (50 μg/lane). α-tubulin served as loading control. (**B**) Densitometric analysis of ORF2p signal intensity. The data (normalized to α-tubulin) are shown as mean ± S.D. of three independent experiments.

We next evaluated the distribution of ORF2p in cancer cell lines of different origin. Protein extracts from several human cancer cell lines (A-375 melanoma, U-87 glioblastoma, HT-29 colon carcinoma, H69 small cell lung carcinoma, BxPC-3 pancreas carcinoma, hormone-sensitive LnCAP and hormone-resistant PC-3, DU 145 prostate carcinoma) were analyzed by immunoblotting using mAb chA1-L1 and compared to human non-immortalized normal WI-38 fibroblasts (Figure 3, panel A). This analysis identified a 150-kDa protein in each cell extract in variable amounts, with the exception of WI-38 cells, which did not exhibit any visible ORF2p expression. Densitometric quantification of the 150-kDa band after normalization to tubulin revealed the highest level of ORF2p expression in A-375 melanoma cells (Figure 3, panel B, histograms). These results suggest therefore that ORF2p expression is a specific feature shared by many tumor cell lines.

### ORF2p is expressed in human colon, prostate, lung and breast tumors but not in their normal tissue counterparts

We wished to assess whether the observed differences between tumorigenic and normal cell lines were reproduced in human bioptic tissues. We selected four types of human neoplasms (Table 1) including i) colon (*n* = 10), ii) prostate (*n* = 54), iii) lung (*n* = 6), and iv) breast (*n* = 4) adenocarcinoma samples (total *n* = 74) and used the chA1-L1 antibody to compare L1-ORF2p expression levels with that of healthy, non-transformed counterparts. We found that 96% of all tumor samples are chA1-L1 immunoreactive, with signal intensities ranging from moderate (+) to very high (+++). In tumor biopsies that stained positive for ORF2p, 30–100% of all examined cells were reactive; no immunoreactivity was appreciated in any of the normal tissues analyzed (Table 1).

**Table 1:** Immunohistochemical analysis of L1-ORF2p expression in human normal and staged cancer tissue samples using mAb chA1-L1

Tissue^1^	Samples	Grade / Gleason score (pattern)	n.	L1-ORF2p positive cells (%)	Signal intensity
**Colon**	Normal mucosa		6	0	–
	Transitional mucosa		10	80	+++
	Adenoma	Low grade	8	50	+
		Intermediate	9	80	++
		High grade	6	90	+++
	Adenocarcinoma		1	30	+
			4	50 – 70	++
			5	80 – 100	+++
**Prostate**	Normal/Hyperplasia		20	0	–/±
	PIN		6	90	++
	Adenocarcinoma	6 (3 + 3)	14	30 – 90	+
		7 (3 + 4); (4 + 3)	23	30 – 90	+
		8–9 (4 + 4); (4 + 5); (5 + 4)	17	30 – 90	+
**Lung**	Normal		8	0	–
	Adenocarcinoma		2	40 – 60	+
			4	70 – 95	++/+++
**Breast**	Normal		7	0	–
	Invasive ductal carcinoma		4	50 – 95	++

Signal intensity: −, same as background; ±, moderately higher than background; +, moderate; ++, high; +++, very high.

1 Staged samples, with their recorded histological information, were enrolled from the repositories or biobanks indicated in Materials and Methods.

**Figure 4:**
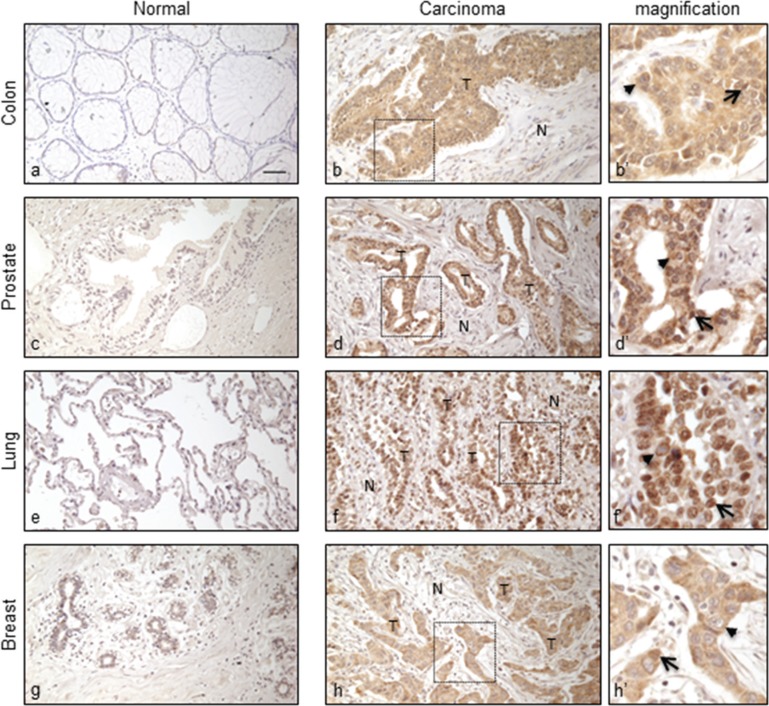
Immunohistochemical staining of ORF2p in human normal and cancer tissue sections Representative tissue sections from: normal colonic mucosa (a), normal prostatic gland (c), normal lung epithelium (e), normal breast (g) and respective carcinomas (b), (d), (f), (h). (N), normal; (T), tumor tissue. Bar, 50 μm; magnification 20x. Boxed areas are shown at a higher magnification in (b′), (d′), (f′) and (h′). Arrow, nuclear localization; arrowhead, cytoplasmic localization.

Figure 4 shows representative ORF2p staining patterns in tumors (panels b, d, f, h) and normal (panels a, c, e, g) tissues; higher magnification (panels b′, d′, f′, h′) indicate that ORF2p is localized both in cell nuclei (arrows) and cytoplasms (arrowheads). It is worth recalling that nuclear localization of L1 proteins has been linked to a poor prognosis in breast cancer [28, 29]. In contrast, no staining was ever detected in the benign areas of the tumor specimens, i.e. striated muscles, adipose and connective tissues. Moreover, we extended the analysis to 27 normal human tissues of different origin (Supplementary Table S1). All analyzed samples were negative, with the exception of skin, heart and thyroid which showed a faint staining. Together, these data confirm that ORF2p overexpression is a distinctive feature of the tumor tissues, in agreement with the results on cancer cell lines.

### ORF2p as a potential early diagnostic marker of tumor onset in colon and prostate

**Figure 5:**
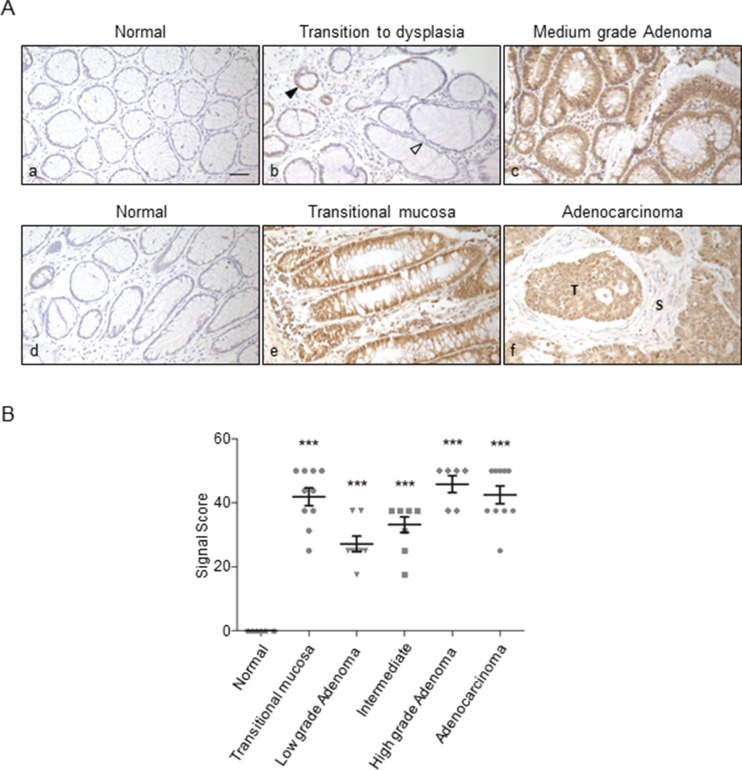
Immunohistochemical staining of ORF2p in human colon tissue sections (**A**) Representative tissue sections from: (a), (d) normal mucosa; (b) transition zone from normal (white arrowhead) to dysplastic mucosa (black arrowhead); (c) adenoma with medium grade dysplasia; (e) transitional mucosa; (f) adenocarcinoma; (T) indicates tumor cells expressing ORF2p, stroma adjacent to the tumor is indicated with (S). Bar, 50 μm; magnification 20x. (**B**) Scatter plot showing distributions and mean values ± SEM of signal scores of colon tissue specimens. Signal scores were assigned to every specimen as described in Materials and Methods; ***P < 0.001.

It was of particular relevance to assess the stage of the lesion at which ORF2p expression is triggered first. To that aim we enrolled a larger cohort of colon and prostate tissue specimens, at various grades of malignant progression, for IHC assays (Table 1). Figure 5A shows representative results of ORF2p staining during colonic mucosa transformation, from normal mucosa (panels a, d) to adenoma (panels b, c) and adenocarcinoma (panels e, f). No immunoreactivity was appreciated in normal mucosa; increasing ORF2p expression was detected in the transition from healthy (panel b, white arrow) to dysplastic mucosa (panel b, black arrow), where expression begins to be visible. High staining intensity was observed in adenoma with medium grade dysplasia (panel c), hyperplastic mucosa adjacent to tumor tissue (panel e, transitional mucosa) and adenocarcinoma (panel f, indicated with T). The results from colon tissue specimens (*n* = 49) are shown in the scatter plot in Figure 5B as distributions and mean (*M*) values ± SEM of signal scores assigned to the specimens (see Materials and Methods for details). Data show a remarkably high mean value for signal scores (*M* = 41. 9) in transitional mucosa, increasing *M* values (from 27. 2 to 45. 8) in adenomas during progression from low to high grade, and high *M* value (*M* = 42. 5) in adenocarcinomas compared to normal tissues (*M* = 0). Thus, ORF2p expression does not only occur in advanced adenocarcinomas (Figure 5A, panel f), but is triggered at very early stages of the tumorigenic process. This conclusion is supported by the finding that both adenoma (Figure 5A, panel c) and transitional mucosa (Figure 5A, panel e) display intense IHC signals. These data further suggest that ORF2p is expressed in a two-wave pattern, with an early sharp wave in the narrow window of transitional mucosa, and a second broader wave from low grade adenoma onwards, which reaches a plateau in high grade adenoma and adenocarcinoma stages.

**Figure 6:**
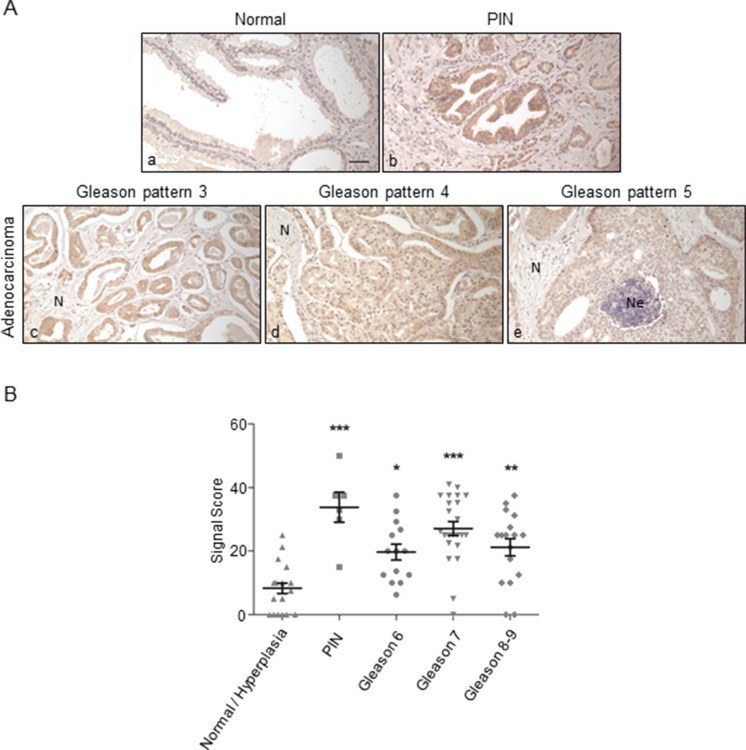
Immunohistochemical staining of ORF2p in human prostate tissue sections (**A**) Representative tissue sections from: normal gland (a); prostatic intraepithelial neoplasia (PIN) (b); adenocarcinoma with Gleason pattern 3 (c), 4 (d) and 5 (e); intraluminal necrotic cells are indicated with (Ne). (N), normal tissue; bar, 50 μm; magnification 20x. (**B**) Scatter plot showing distributions and mean values ± SEM of signal scores of prostatic tissue specimens. *P < 0.05; **P < 0.01; ***P < 0.001.

Parallel results were obtained from the analysis of prostate carcinoma samples (Figure 6A): ORF2p was abundantly expressed in prostatic intraepithelial neoplasia (PIN), a precancerous lesion (panel b) that showed signals higher than adenocarcinomas with Gleason pattern 3 (panel c), 4 (panel d) or 5 (panel e). In normal prostatic gland (panel a) no immunoreactivity was detected; weak staining (±) was occasionally observed on hyperplastic epithelia.

Figure 6B summarizes the IHC staining results from prostate tissue specimens (*n* = 80) in a scatter plot. Tissue samples were grouped according to histological features: Gleason grading of specimens (from 6 to 9) [43] derives from the sum of primary and secondary Gleason patterns. The PIN group shows the highest mean value of signal scores (*M* = 33,8), compared to Gleason 6, 7 and 8–9 adenocarcinomas (*M* = 19. 7, 27. 2 and 21. 2, respectively) and to normal/hyperplasia tissues (*M* = 8. 3). Results are summarized in Table 1. Interestingly, besides high level of ORF2p expression in PIN, prostate adenocarcinoma tissues exhibited pronounced heterogeneous staining intensities among the histological groups. On the whole, these data strongly suggest that overexpression of endogenous ORF2p occurs at a very early stage of prostate transformation and confirm that the bimodal pattern of ORF2 expression also characterizes prostate cancer progression.

## DISCUSSION

Growing data in the last few years confirm that cancer cells and tissues offer a highly permissive environment for the expression and mobilization of L1 retrotransposons. Numerous reports have consistently shown that the genomes of different cancer types harbor hundreds of *de novo* somatic insertions, which were found to be selectively present in tumor genomes [31, 44–48]. It has been debated, however, whether L1 retrotransposition events are “drivers” with a causative role in tumorigenesis, or passive “passengers”, representing a consequence of the loss of genome regulation associated with cell transformation [12]. The finding that RT inhibition has anti-proliferative and differentiative effects in cancer cells [14, 22], and antagonizes cancer progression in murine models [14], supports a tumor-promoting role of L1-encoded RT activity.

In that framework, it was mandatory to evaluate accurately the expression of L1-ORF2 RT in cancer tissues, since no systematic study had ever been carried out to this purpose. Here we have developed a highly specific monoclonal antibody, chA1-L1, raised against a peptide in the human ORF2p EN domain, the same domain that has been already targeted by others to generate both polyclonal and monoclonal anti-L1-ORF2p antibodies [49, 50]. The antibody has enabled us to reveal L1-ORF2p expression in cancer cell lines and bioptic tissues from staged carcinomas. Several interesting findings were uncovered. First, immunoblot analysis shows that ORF2p is widely overexpressed in human cancer cell lines compared to normal human fibroblasts, confirming the link between ORF2p expression and tumorigenesis; U87-MG glioblastoma is an exception being the only cell line with barely detectable ORF2p (Figure 3), in good agreement with results reporting that also ORF1p is expressed at low levels in brain tumors [27]. Second, immunohistochemical analyses of colon, prostate, lung and breast bioptic tissues confirmed high ORF2p expression in carcinoma samples but not in their healthy counterparts (Figure 4), consistently with a previous study demonstrating the expression of ORF2p in breast cancer [29]. Similarly to our results on breast, lung, prostate and colon, Rodic et al. [27] had reported that ORF1p is also variably expressed in advanced neoplasms of different origin although ORF1p was not frequently detected in preneoplastic lesions. However, it is possible that this discrepancy might be caused by differential stability of the two ORF proteins.

Indeed, high ORF2p expression levels were observed at very early transformation stages in colon and prostate specimens, before the appearance of characteristic histological features of carcinoma (Figures 5 and 6). Interestingly, precancerous lesions, such as transitional colonic mucosa (Figure 5, panel e) and prostate intraepithelial neoplasia (PIN) (Figure 6, panel b), show significantly intense signals compared to controls (*P* < 0.001). Together, these data parallel other evidences demonstrating that genomic hypomethylation is an early event in colon tumorigenesis [51] and that in prostate carcinoma L1 methylation decreases in premalignant PIN and declines further throughout tumor progression [52]. Moreover, the levels of L1 hypomethylation are also highly variable among different cases of prostate cancer [53], a finding that could explain the pronounced variability of L1-ORF2p expression observed in the different adenocarcinoma specimens (Figure 6). The recent finding that L1 expression and retrotransposition are triggered in both Barrett's esophagus and esophageal carcinoma [31] strengthens our observations of ORF2p premalignant expression in colon and prostate tissues. Since retrotransposons are known to be activated as part of the stress response [54], it is possible that the early upregulation of ORF2p in colon transitional mucosa and PIN is a response to stress conditions associated with the tumor microenvironment (TME).

In staged samples from both cancer types analyzed in depth here, ORF2p appears to be stimulated in a bimodal pattern, with a sharp initial burst at a very early stage, followed by a second steady moderate wave throughout the latest stages. In our opinion, the present finding that L1-ORF2p expression precedes tumorigenesis further supports the view that high L1 activity could be a trigger of cell transformation rather than its consequence. In this context, early ORF2p expression could be regarded as a preneoplastic marker, at least in colon and prostate. Further work will establish whether ORF2p expression is a widespread early sign of transformation in diverse cancer types.

In breast cancer studies, using murine models [26] and human samples [29], ORF2p translocation from the cytoplasm to nuclei was observed in advanced stages; the nuclear localization would be consistent with the massive occurrence of retrotransposition events, requiring ORF2p-associated RT activity and thought to contribute to shape the “cancer genome” [55]; this model would account for the second broad wave of ORF2p expression. The function of early ORF2p overexpression remains to be elucidated. Studies of breast cancer progression in mouse models revealed L1 copy number amplification in early stages, not necessarily followed by insertions [26]. It might be speculated that the two waves correspond to distinct steps in cancer progression: i) the normal-to-precancerous transition, and ii) the evolution from the latter to overt cancer. Aberrant activation of the L1-RT mechanism would induce cell transformation by sequentially converting normal to preneoplastic and eventually to cancer cells through these subsequent steps, suggesting a key role of the RT enzyme in both phases. In summary, the present data indicate that ORF2p expression represents a potentially valuable early diagnostic biomarker and identify the monoclonal antibody chA1-L1 as a useful tool for both basic cancer studies and diagnostic applications.

## MATERIALS AND METHODS

### Generation of monoclonal antibody chA1-L1 against human L1-encoded ORF2p

To raise mouse monoclonal antibodies against human L1-ORF2p, the amino acid sequence of L1_RP_-ORF2p (accession number S65824) [33] was aligned with the murine L1_spa_-ORF2p sequence (accession number AAC53542.1) [56] using Vector NTI^®^ software (Life Technologies), in order to identify regions with the lowest homology (below 40%) between human and mouse L1 sequences. Six human peptides were selected:

#39, aa 119–138:TGAPRFIKQVLSDLQRDLDS;
#40, aa 231–248:SAIKLELRIKNLTQSRST;
#41, aa 745–765:NNRQTESQIMGELPFVIASKR;
#42, aa 945–962:RKLKLDPFLTPYTKINSR;
#43, aa 980–1000:NLGITIQDIGVGKDFMSKTPK;
#44, aa 1021–1044:TAKETTIRVNRQPTTWEKI FATYS.

Peptides were synthesized, coupled to Keyhole Limpet Hemocyanin (KLH) (Eurogentec, Belgium) and used to immunize BALB/c mice (Charles River, Germany). Mice were maintained at the Paul-Ehrlich-Institute, Langen, Germany, in accordance with the institutional directive on laboratory animal welfare. All animal experiments were approved (Permit F107/111) by the local authorities and performed according to ethical principles. Mice sera were tested for reactivity against all six immunogenic peptides by indirect ELISA assays and used in immunoblot analyses to test for the ability to detect proteins with a molecular weight (MW) of ∼150 kDa (theoretical MW of L1-ORF2p) in human 2102Ep embryonal carcinoma cell (ECACC: N2102Ep clone 2/A6) lysates. Mice with both high and specific humoral immune response were selected for splenectomy and the obtained B-lymphocytes were fused with X63Ag8.653 murine myeloma cells [57] (kindly provided by K. Boller, Paul-Ehrlich-Institute), in the presence of polyethylene glycol. Hybrid cell supernatants were screened for immunoreactivity by ELISA and immunoblot assays. One specific hybridoma clone, obtained from a mouse immunized with peptide 39, was finally chosen for single-cell cloning and the released monoclonal antibody (mAb), referred to as chA1-L1 (isotype IgG2a), was purified using the Pierce Thiophilic Adsorption kit (Thermo Scientific, USA).

### ELISA assay

96-well MaxiSorp plates (Nunc/Thermo Scientific, USA) were coated with 100 ng/well of each peptide; negative control wells were coated with BSA or a scrambled peptide; KLH was used as a positive control. Plates were blocked with 3% BSA / 10% fetal bovine serum in PBS, then incubated with mice sera diluted in blocking solution. To detect bound IgG antibodies, an HRP-conjugated goat anti-mouse IgG antibody (1:20000; Abcam, UK) was added to each well. After addition of the substrate solution (o-phenylenediamine dihydrochloride, Sigma-Aldrich), the reaction product was quantified by light absorption (490 nm wavelength).

### Cell cultures

A-375 melanoma cells (ATCC CRL-1619), U-87 MG glioblastoma cells (ATCC HTB-14), A-375 melanoma cells stably interfered for L1 expression (pS-L1i) and their control cell line (stably transfected with non-interfering vector pS-neo) [described in 16] were cultured in DMEM (Lonza). Non-transformed WI-38 human lung fibroblasts (ATCC CCL-75) were cultured in EMEM (Lonza) with 1% non-essential amino acids. HT-29 colon carcinoma (ATCC HTB-38), H69 small cell lung carcinoma (ATCC HTB-119), BxPC-3 pancreatic carcinoma (ATCC CRL-1687), PC-3 (ATCC CRL-1435), LNCaP (ATCC CRL-1740) and DU 145 (ATCC HTB-81) prostate carcinoma cell lines were cultured in RPMI 1640 medium (Gibco). Media were supplemented with 10% FBS, 2 mM L-glutamine and 1x penicillin/streptomycin (Lonza). Cell lines purchased from ATCC were cultured immediately upon receipt, propagated for few passages and frozen as aliquots; when needed, cells were subcultured for no more than one month, to maintain a low number of population doublings. All cell lines were routinely tested for *Mycoplasma* contamination using the Venor-GeM Mycoplasma kit (Sigma-Aldrich).

### Plasmids and cell transfection

The pTT5-L1 plasmid, encoding codon-optimized L1-ORF2 which is expressed under the control of the CMV promoter and cloned between EcoRI and HindIII restriction sites (Supplementary Figure S1), was generated commercially (GenScript) and kindly provided by Alienor Farma (Bordeaux, France). The pCMV-hGH plasmid [35] codes for the human growth hormone (hGH) and is under transcriptional control of the CMV promoter. A-375 melanoma cells were seeded 24 hours before transfection and transfected with plasmids pTT5-L1 or pCMV-hGH using FuGENE HD reagent (Promega). 48 hours post-transfection cells were harvested for protein extraction or fixed for immunofluorescence assays. FuGENE HD only-treated A-375 cells (Mock) were used as a negative control.

### Protein extraction and immunoblot analyses

8 × 10^6^ cells were lysed in M-PER buffer (Thermo Scientific) supplemented with protease inhibitor cocktail (Roche). 50 μg of whole cell extract were used for immunoblotting as previously described [26]. mAb chA1-L1 (0.7 μg/ml) was used as the primary antibody. For the peptide competition assay, membranes were probed with an equimolar mixture of chA1-L1 and peptide 39, pre-incubated for 45 minutes at room temperature. The sensitivity of the antibody was assessed by immunoblot analysis of increasing amounts (1 to 75 ng) of the purified L1 endonuclease domain (L1-EN) [34] added to 50 μg of WI-38 whole cell extracts. HRP-conjugated goat anti-mouse IgG (Abcam) was used as the secondary antibody. Membranes were probed with an anti-α-tubulin antibody (Sigma-Aldrich) to verify equal loading.

### Immunofluorescence assays

5 × 10^5^ A-375 melanoma cells were grown on sterile coverslips in 6-well plates. Where indicated, cells were transfected as described above and fixed in cold methanol. Slides were blocked in 3% BSA, 0.05% Tween20 in PBS, 30 minutes at 37°C, then incubated with mAb chA1-L1 alone or in combination with a rabbit polyclonal anti-ORF1p antibody [38] diluted in blocking solution (8 μg mAb/ml) for 1 hour at 37°C. FITC-conjugated goat anti-mouse IgG + IgM and Cy3-conjugated donkey anti-rabbit IgG (Jackson Immunoresearch) were used as secondary antibodies. Samples were stained with DAPI, mounted in Vectashield (Vector) and visualized under a Nikon Eclipse 90i microscope. Images were captured along the z-axis and deconvolved using the NIS-Elements software (Nikon) at the Nikon microscopy reference center at the Institute of Molecular Biology and Pathology, CNR, Rome.

### Immunohistochemical (IHC) analyses

Human bioptic tissue specimens were obtained from IRCCS San Raffaele Interinstitutional Multidisciplinary BioBank (BioBIM) (Rome, Italy) and Istituto Nazionale Tumori Regina Elena (National Cancer Institute, Rome, Italy) or purchased as tissue microarrays (Biochain, Newark, CA, USA; Super Bio Chips, Seoul, Korea; Cybrdi Inc, Rockville, MD, USA). Informed consent was obtained from subjects enrolled in this study; all investigations were performed in accordance with ethical principles embodied in the Declaration of Helsinki.

Formalin-fixed paraffin-embedded human tissue sections and microarrays were deparaffinized, rehydrated in descending graded ethanol solutions and treated with 0.6% hydrogen peroxide in methanol. Heat-induced antigen retrieval was achieved in Citrate buffer, pH 6.0 (Novus Biologicals, UK). Slides were pre-incubated with Protein Block reagent (Abcam, UK) for 1 hour at room temperature, and subsequently incubated overnight at 4°C with chA1-L1 mAb (8 μg/ml) in 1% BSA/PBS. Sections were stained using the Mouse Specific HRP/DAB (ABC) kit (Abcam), counterstained with Hematoxylin (Sigma-Aldrich), dehydrated in ethanol, mounted in Eukitt medium (Sigma-Aldrich) and visualized under a light microscope (Leica Microsystems) equipped with a cooled camera (SPOT RT3, SPOT Imaging Solutions) and the IAS 2000 software for image capture.

### IHC signal evaluation and scoring

In an investigator-blind study, two researchers independently evaluated the stained human tissue sections. The percentage of ORF2p-positive cells (mAb chA1-L1 stained / total cells) was calculated after scoring an average of five fields at 20x magnification; for overt carcinomas, histologically benign areas were excluded from the scoring. Signal intensities were classified as follows: –, same as background; ±, moderately higher than background; +, moderate; ++, high; +++, very high. Colon and prostate specimens were grouped according to histological features and signal intensities were scored (arbitrary units from 0 to 50) according to the following criteria: –, score 0; ±, score 10; +, score 25; ++, score 37,5; +++, score 50. Scatter plots show distributions and mean (M) values ± SEM of assigned signal scores; signal intensities corresponding to mean values are further indicated in Table 1. Statistically significant differences were evaluated using the one-way ANOVA test and analyzed in Multiple Comparisons versus Control Group (Tukey's test, *P* < 0.05); statistical analyses were performed using the GraphPad Prism software.

## SUPPLEMENTARY MATERIAL FIGURES AND TABLE


