# Supplementary material for: Enhanced expression of LINE-1-encoded ORF2 protein in early stages of colon and prostate transformation
Source: Oncotarget. 2015 Dec 26;7(4):4048–61. doi: 10.18632/oncotarget.6767 (PMC4826189; doi:10.18632/oncotarget.6767)
Supplement: Supplementary file 1 [file oncotarget-07-4048-s001.pdf]

Enhanced expression of LINE-1-encoded ORF2 protein in early stages of colon and prostate transformation

Supplementary Materials

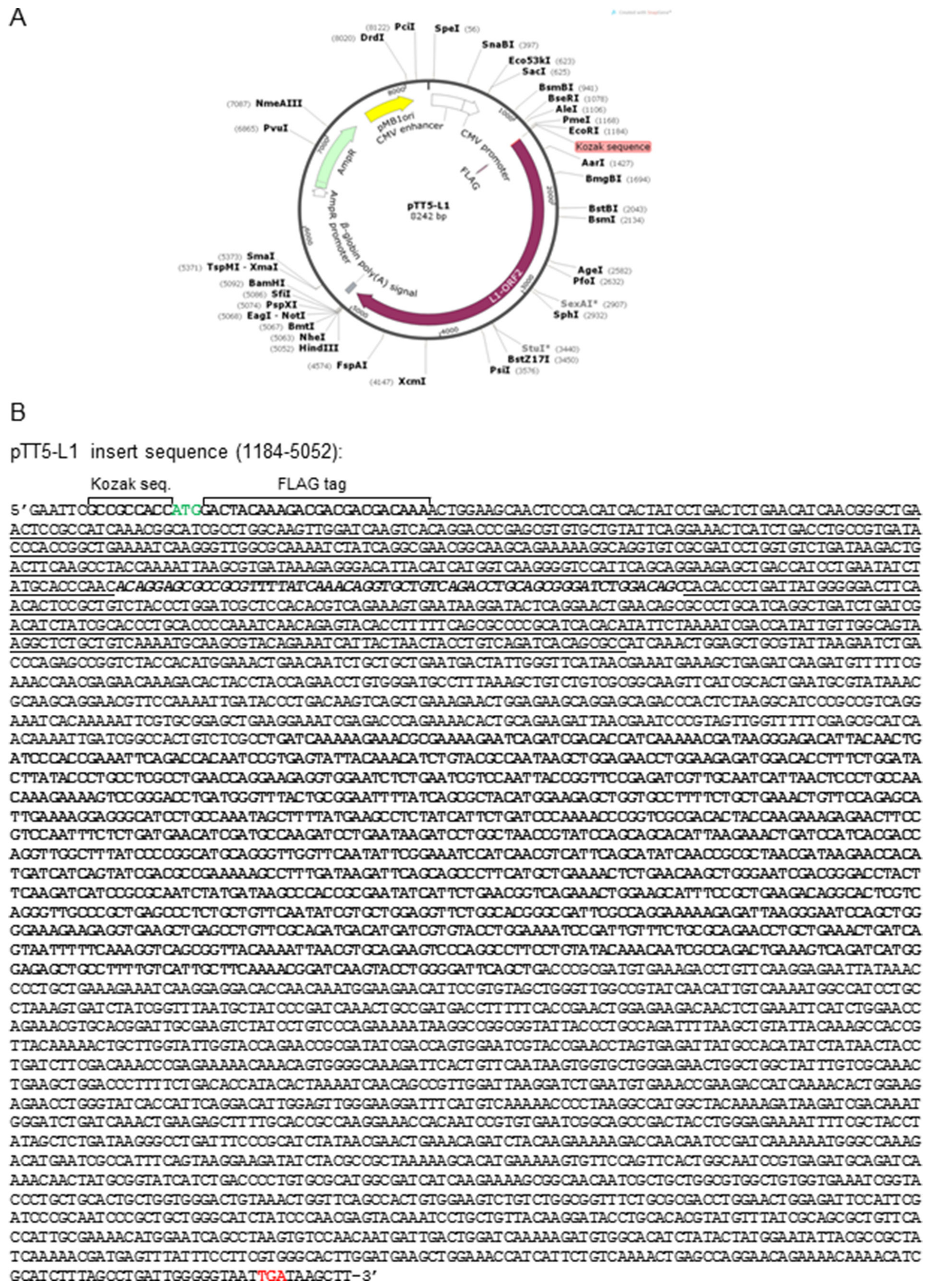

Supplementary Figure S1: pTT5-L1 plasmid map. (**A**) Structure and (**B**) sequence of the insert coding for ORF2p; coding sequence of pep. 39 (bold italic lettering).

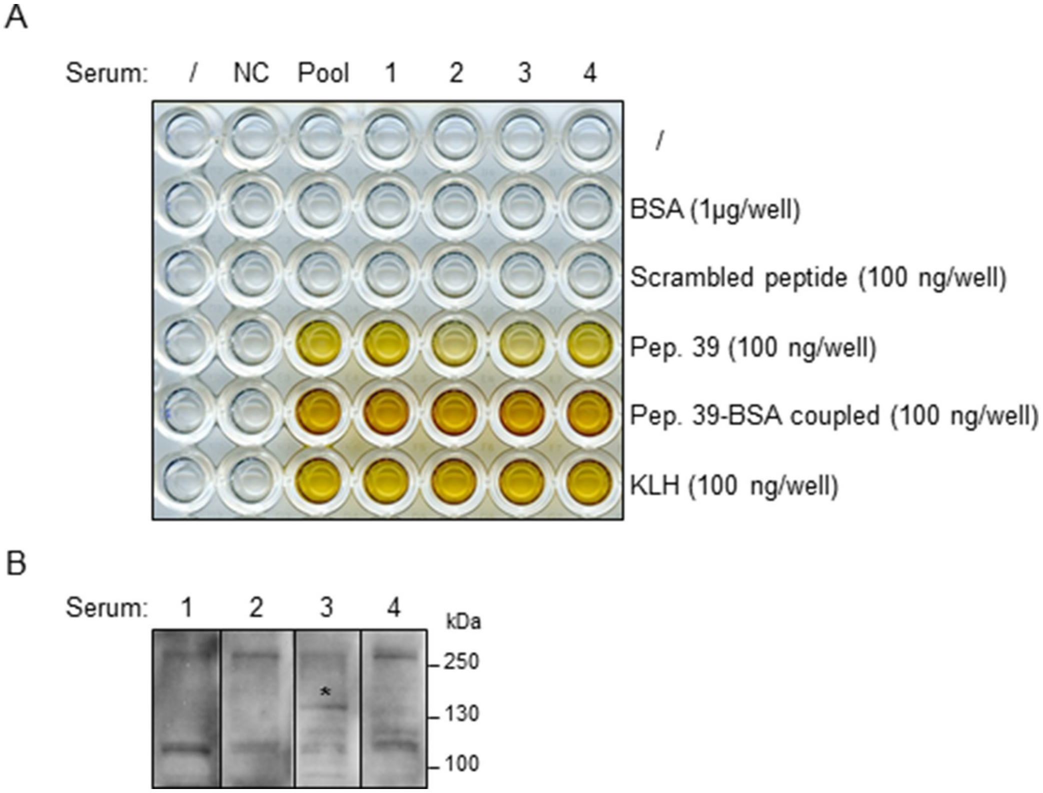

Supplementary Figure S2: Analysis of the production and specificity of the anti-peptide 39 antibodies. (**A**) ELISA assay on sera from 4 mice (#1, 2, 3, 4) immunized with peptide 39, a negative control mouse (NC) and a pool of sera from immunized mice (pool). Wells were coated as described on the right. (**B**) Immunoblot reactivity analyses of sera from mice #1, 2, 3, 4 on whole cell extracts from human 2102Ep embryonal carcinoma cells. Asterisk indicates the detection of a 150-kDa protein, putative L1-encoded ORF2p.

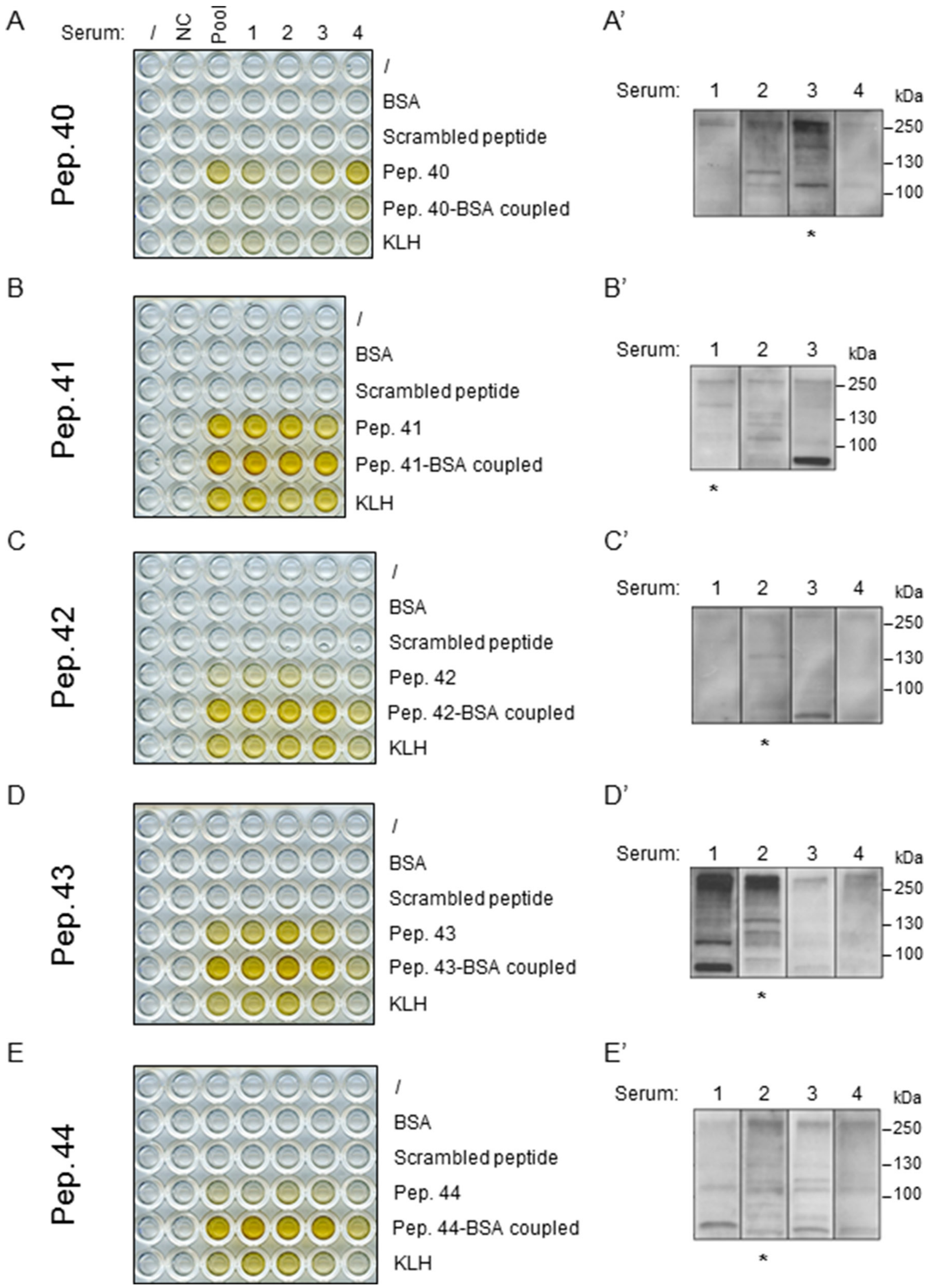

Supplementary Figure S3: Analysis of the production and specificity of the antibodies raised against peptides 40 to 44. Sera from groups of 4 mice (#1, 2, 3, 4), each group immunized with one peptide (#40, 41, 42, 43 or 44), were tested by indirect ELISA assays (**A, B, C, D, E**) and used in immunoblot analyses (**A', B', C', D', E'**) to test for the ability to detect proteins with a MW of ~150 kDa (theoretical of ORF2p) in human 2102Ep embryonal carcinoma cell lysates. The presence of an asterisk indicates the detection of a 150-kDa protein , putative L1-encoded ORF2p.

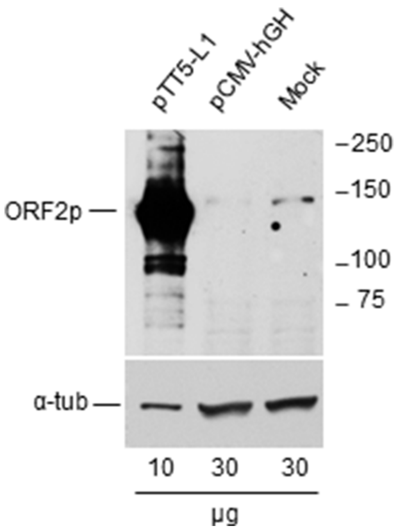

Supplementary Figure S4: Endogenous expression and overexpression of ORF2p in A-375 cells is specifically detected by chA1-L1 antibody. Immunoblot analysis of whole cell extracts from pTT5-L1-, pCMV-hGH- and mock-transfected A-375 cells to detect transiently overexpressed or endogenous ORF2p. Notably, only 10 µg of whole cell extract were loaded for pTT5-L1-transfected A-375 cells (i.e. 3X less than pCMV-hGH- and mock-treated cells); α-tubulin expression served as a loading control (lower panel).

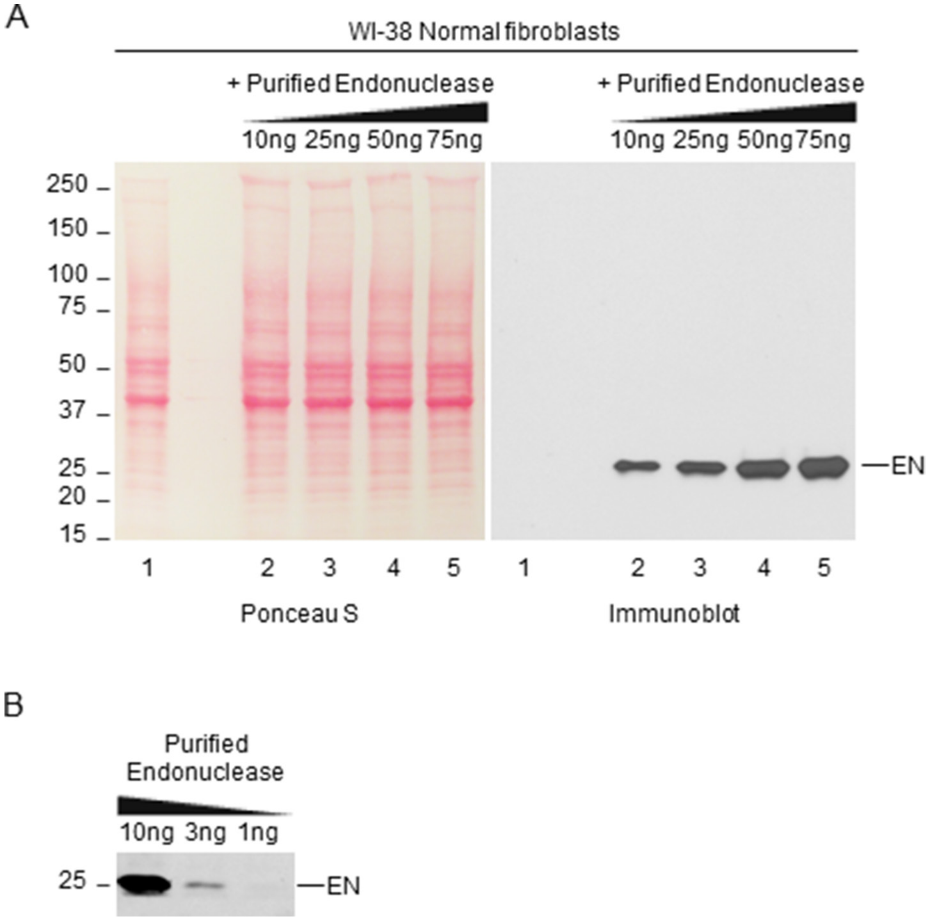

Supplementary Figure S5: Sensitivity assay of the chA1-L1 monoclonal antibody. (**A**) Whole cell extract (50 µg/lane) from WI-38 human normal fibroblasts was pre-mixed with bacterially expressed L1-Endonuclease (EN) at the amounts indicated (lanes 2–5), loaded on SDS-PAGE and stained with Ponceau S (left panel); filter was then incubated with chA1-L1 mAb (right panel) and exposed for 3 seconds to a light sensitive X-ray film. As a negative control, extract from WI-38 cells was loaded in lane 1. (**B**) To further assess chA1-L1 mAb sensitivity, we scaled down the amount of purified endonuclease to 1 ng per lane. Filter was exposed for 1 minute to a light sensitive X-ray film.

Supplementary Table S1: Immunohistochemical staining of human normal tissues using chA1-L1 monoclonal antibody

Tissue	*n.*	Signal intensity
Aorta	2	−
Bladder	3	−
Bone marrow	1	−
Cerebellum	5	−
Cerebrum	7	−
Eye	1	−
Esophagus	5	−
Small intestine	5	−
Rectum	3	−
Fallopian tube	3	−
Heart	5	±
Lymph node	2	−
Ovary	3	−
Pancreas	5	−
Peripheral nerve	2	−
Pituitary gland	2	−
Skin	3	±
Spinal cord	2	−
Spleen	4	−
Striated muscle	5	−
Subcutis (adipocytes)	2	−
Thymus	5	−
Thyroid	4	±
Tonsil	5	−
Umbilical cord	2	−
Ureter	3	−
Uterus	7	−
